# Selective 5HT3 antagonists and sensory processing: a systematic review

**DOI:** 10.1038/s41386-021-01255-4

**Published:** 2022-01-11

**Authors:** Eirini Tsitsipa, Jonathan Rogers, Sebastian Casalotti, Clara Belessiotis-Richards, Olga Zubko, Rimona S. Weil, Robert Howard, James A. Bisby, Suzanne Reeves

**Affiliations:** 1 Division of Psychiatry, University College London, 149 Tottenham Court Road, London, W1T7NF UK; 2 South London and Maudsley NHS Foundation Trust, Bethlem Royal Hospital, Monks Orchard Road, Beckenham, BR3 3BX UK; 3 Dementia Research Centre, University College London, 8-11 Queen Square, London, WC1N 3AR UK; 4 Wellcome Centre for Human Neuroimaging, University College London, 12 Queen Square, London, WC1N 3AR UK; 5 Movement Disorders Consortium, National Hospital for Neurology and Neurosurgery, Queen Square, London, WC1N 3AR UK

**Keywords:** Perception, Predictive markers

## Abstract

Ondansetron is a selective serotonin (5HT3) receptor antagonist that is under evaluation as an adjunctive treatment for schizophrenia, and a novel treatment for hallucinations in Parkinson’s disease. Ondansetron reverses sensory gating deficits and improves visuoperceptual processing in animal models of psychosis, but it is unclear to what extent preclinical findings have been replicated in humans. We systematically reviewed human studies that evaluated the effects of ondansetron and other 5HT3 receptor antagonists on sensory gating deficits or sensory processing. Of 11 eligible studies, eight included patients with schizophrenia who were chronically stable on antipsychotic medication; five measured sensory gating using the P50 suppression response to a repeated auditory stimulus; others included tests of visuoperceptual function. Three studies in healthy participants included tests of visuoperceptual and sensorimotor function. A consistent and robust finding (five studies) was that ondansetron and tropisetron (5HT3 antagonist and α7-nicotinic receptor partial agonist) improved sensory gating in patients with schizophrenia. Tropisetron also improved sustained visual attention in non-smoking patients. There was inconsistent evidence of the effects of 5HT3 antagonists on other measures of sensory processing, but interpretation was limited by the small number of studies, methodological heterogeneity and the potential confounding effects of concomitant medication in patients. Despite these limitations, we found strong evidence that selective 5HT3 antagonists (with or without direct α7-nicotinic partial agonist effects) improved sensory gating. Future studies should investigate how this relates to potential improvement in neurocognitive symptoms in antipsychotic naive patients with prodromal or milder symptoms, in order to understand the clinical implications.

## Introduction

Serotonin (5-HT) is a biogenic monoamine with a complex role in regulation of the sleep-wake cycle, appetite, pain, body temperature, vomiting, cognitive, perceptual and affective functions [1]. The involvement of 5HT in hallucinations was first established in clinical experiments with psychedelics (5HT2A agonists such as psilocybin and lysergic acid diethylamide) which are associated with heightened and or altered sensory experiences, including visual and auditory hallucinations [2]. Subsequent research has shown a prominent role of 5HT in early sensory processing, mediated through serotonergic innervation of primary sensory cortices, and subcortical structures (including the amygdala and thalamus) that modulate neuronal responses to sensory stimuli [3, 4]. The antipsychotic potential of serotonergic agents has been of longstanding interest [5], since the discovery that clozapine, whose multi-target action includes 5HT1A, 5HT2A/C and 5HT3 receptors, was effective in reducing psychotic symptoms in patients with treatment-resistant schizophrenia. Research in this area has been largely directed towards schizophrenia, but the focus has recently shifted towards psychosis in the context of neurodegeneration, encouraged by pivotal trials of the 5HT2A receptor partial inverse agonist Pimavanserin, which showed modest benefits in the treatment of Parkinson’s disease (PD) psychosis [6, 7].

There are multiple potential serotonergic agents of interest, including the 5HT3 receptor antagonist ondansetron, that is licensed for use as an anti-emetic and under evaluation in the phase II Trial of Ondansetron as a Parkinson’s Hallucinations Treatment (TOP HAT) (Clinical trials.gov NCT04167813). 5HT3 receptors are cation selective ion channels, closely related to nicotinic acetylcholine receptors, and are highly expressed in mesocorticolimbic regions that are involved in sensory information processing, and assign context and salience to sensory inputs [8]. Distinct from other 5HT receptor subtypes, whose actions are G-protein coupled, 5HT3 receptors mediate fast synaptic neurotransmission and modulate the release of multiple neurotransmitters (dopamine, substance P, gamma-aminobutyric acid (GABA) and acetylcholine) through their expression on pre-synaptic terminals of non-serotonergic neurones [9, 10] and post-synaptic GABAergic inter-neurones [11]. Although there is no evidence of a direct involvement of 5HT3 receptors in hallucinations, preclinical studies have shown antipsychotic and pro-cognitive effects of 5HT3 receptor antagonist ondansetron in animal models that are relevant for psychosis in humans [12]: Ondansetron attenuated amphetamine-induced dopamine release in mesocorticolimbic regions without inducing parkinsonism [9, 10, 13]; and reversed impairments in sensory gating and visual processing in the DBA/2 mouse model of α7-nicotinic cholinergic receptor depletion, by increasing hippocampal acetylcholine release [14, 15]. The latter findings extend to the second-generation 5HT3 antagonist tropisetron; a selective 5HT3 antagonist with additional partial α7-nicotinic receptor agonist activity [16]. In vitro demonstration that many antidepressant and antipsychotic drugs functionally antagonise 5HT3 receptors in a non-competitive way, by inhibiting 5HT-induced ion influx, and that clozapine and olanzapine have direct 5HT3 receptor antagonist effects, provided additional, indirect evidence of the involvement of 5HT3 receptors in psychotropic drug action [17, 18].

Clinical evidence supportive of ondansetron’s potential efficacy in the treatment of Parkinson’s hallucinosis was shown in the early 1990s in a case series of 7 inpatients (5 men, aged 64–78 years) all of whom responded to ondansetron 12–20 mg/day without side effects, and with complete symptom resolution in 4 patients [19]. An open study carried out by the same research group, included 16 patients (11 men, aged 64–68 years) with Parkinson’s and severe, persistent visual hallucinations, who were titrated from 4–8 mg/day ondansetron to an optimum dose (mean 16 mg/day, range 12–24 mg/day), and evaluated after 4–8 weeks’ treatment. There were marked improvements in visual hallucinations (complete symptom resolution in 14), and associated delusions (partial to complete response in 8 of 9), with no worsening of motor symptoms or cognition, and improved global functioning [20]. Although the drug has been used off-licence by some clinicians, this line of development was not pursued by the manufacturer, and the previously high cost of ondansetron prevented further independent studies. Since coming off patent in 2006, ondansetron has been evaluated in phase II trials as an adjunctive treatment for patients with schizophrenia who were either treatment refractory or symptomatically stable on antipsychotic medication. These studies have shown evidence of efficacy in the treatment of both negative and cognitive symptoms, including some evidence of improved visual information processing [21–23], and the drug is under phase II evaluation in a Trial of Ondansetron as a Parkinson’s HAllucinations treatment (TOP HAT) (Clinical trials.gov NCT04167813). The observation that ondansetron and other 5HT3 antagonists (tropisetron, granisetron, dolasetron) improve the ability to filter (gate) irrelevant stimuli and enhance visual information processing in animal models, is of potential therapeutic relevance as sensory gating deficits are a robust neurophysiological marker of psychosis in humans [24–26]. We systematically reviewed human studies that had evaluated the effects of ondansetron and related compounds on sensory gating or sensory processing, in both clinical populations and in healthy participants.

## Methods

### Study design

We conducted a systematic review with narrative synthesis, which followed the Preferred Items for Reporting of Systematic Reviews and Meta-Analyses (PRISMA) guidelines [27] and aimed to determine the effect of 5HT3 receptor antagonists on sensory processing.

### Eligibility criteria

We included human studies of healthy participants and any clinical population to identify the impact of 5HT3 antagonists on sensory (visual, olfactory, auditory, gustatory, tactile) or sensorimotor function. Studies were included if participants were administered a 5HT3 receptor antagonist (ondansetron, palonosetron, tropisetron, dolasetron, granisetron) and compared to those not taking a 5HT3 antagonist. We included studies if their primary or secondary outcomes involved evaluation of sensory or sensorimotor functioning, comprising investigations of auditory gating deficits, visuoperceptual functioning and any neuroimaging study outcomes. Studies were excluded if they evaluated the impact of 5HT3 antagonists on pain or pruritus. We included randomized control trials (RCT), cohort studies, and controlled clinical studies, and excluded protocols, case studies, dissertation theses, meeting abstracts, unpublished dissertations, and conference presentations. Only studies written in English were included.

### Search strategy

A database search was carried out in August 2021, using Medline, Embase, PsycINFO and Web of Science (WoS). Relevant reviews and references of the included studies were searched manually to identify appropriate studies for this review. Databases were searched using the following terms:

Ondansetron OR Zofran OR Zophren OR tropisetron OR granisetron OR Sancuso OR Kytril OR “Apo-Granisetron” OR Kevatril OR Sustol OR dolasetron OR Anzemet OR Anemet OR Zamanon OR palonosetron OR Aloxi OR Akynzeo OR Jiouting OR Onicit OR Palnox OR Paloxi OR Palzen OR Themiset OR Zhiruo OR “5-ht3 antagonist” OR “5-ht3R”) AND (visuopercept* OR visuospat* OR visual memor* OR visual recog* OR sensor* OR olf* OR gust* OR tact* OR propriocept* OR interocept* audit*).

The search terms were differentiated based on the variable databases and their respective search criteria, as the subject headings are different (see supplementary materials for the full search strategy). Two authors (SR and ET) independently and blindly screened all the titles and abstracts against the eligibility criteria. Full texts of the remaining studies were assessed against the eligibility criteria (SR and ET). Any disagreements were resolved through discussion between these two authors.

### Data extraction

ET extracted data from all the studies, with JR, CBR and SC acting as second blind raters. Any disagreements were resolved with discussion between two raters and any unresolved differences were discussed with JAB. Effect measures were reported as recorded by the study authors. Data were extracted in regard to the relevant outcomes (any observed effects of 5HT-3 receptor antagonists on sensory or sensorimotor functioning), study characteristics (design, setting, population, inclusion/exclusion criteria) and clinical characteristics (population, sample size, diagnostic criteria, age, sex). All relevant outcomes were recorded.

### Risk of bias in individual studies and quality assessment

The Quality Assessment Tool for Quantitative Studies developed by the Effective Public Health Practice Project (EPHPP) was used to assess bias [28]. This tool is designed to evaluate several study designs and assesses the following: selection bias, study design, confounders, blinding, data collection methods, withdrawals and dropouts, intervention integrity and analysis. The scores in the subsections were used to generate an overall rating of ‘strong’, ‘moderate’ or ‘weak’ using the EPHPP guidance, with a ‘strong’ paper defined as that with no ‘weak’ subscores, and a ‘weak’ paper having two or more ‘weak’ subscores. ET completed EPHPP for all the included studies whilst CBR, JR and SC independently and blindly rated the quality of the studies. Any differences were solved with discussion and any unresolved discrepancies were discussed with JAB.

### Synthesis of results

Due to the small number and heterogeneity of the studies, we carried out a narrative synthesis. All included studies were presented in a table, listing the extracted variables.

## Results

### Study identification

Of the 12 studies initially identified, two papers [29, 30] described an identical sample and, on further exploration, were found to report the same trial, 1 year apart (one reporting 12 weeks data; another data from the first 8 weeks). Data were extracted from the paper reporting 12 weeks of data, and the other paper [30] was excluded. The PRISMA flowchart is shown in Supplementary Fig. 1.

### Study characteristics

Reviewed studies included five randomised control trials with parallel group design [29, 31–34], five randomised control trials with crossover design [35–39] and one cohort (patients versus healthy controls) study [40]. The age of participants ranged from 19 to 55 years. Studies were conducted in the UK, USA, China, Israel, Japan and Iran. Of the five crossover studies, three included healthy volunteers [36, 37, 39], one involved participants with schizophrenia stable on unspecified antipsychotics [35], and one assessed participants with schizophrenia stable on clozapine [38]. The parallel group studies all included patients with schizophrenia, whose symptoms were stable on risperidone. Six studies evaluated ondansetron [33, 35, 36, 38, 39], four evaluated tropisetron [31, 32, 34, 40] and one granisetron [37]. Characteristics and details of the included studies are described in Table 1.

**Table 1:** Study characteristics and findings.

Author, year Country Type of study	Population and referral setting	Intervention, sample size (*N*), duration	Mean age ± SD years Number (%) females	Outcome measure(s) of interest	Type of statistical analysis	Summary of findings
Adler, 2005 USA Double-blind placebo-controlled crossover trial	Diagnosis of schizophrenia, based on PANSS and BPRS scores, stable on antipsychotic medication NR	Randomised in terms of order: A. Ondansetron 16 mg + placebo B. Placebo + ondansetron 16 mg *N* = 8, Single dose	42 ± 6 4/8 (50%) female	Primary Outcome: P50 auditory evoked potential, assessed using the paired-click paradigm (500 ms apart), and indexed by the T/C ratio.	Parametric and nonparametric methods. A priori comparison made for T/C ratio after 2 h (peak plasma concentration) using two-tailed Student’s *t* test. Other *t* tests were Bonferroni corrected.	Ondansetron associated with improvement in P50 auditory gating (*t*_10,7_ = −2.43, *p* < 0.04]) Seven patients who were treated with ‘typical’ antipsychotics showed significant change, and no improvement was observed in one olanzapine-treated patient
Akhondzadeh, 2009 Iran Double-blind placebo-controlled parallel group trial	Diagnosis of schizophrenia (DSM-IV-TR), stable on risperidone (4–6 mg/day) for a min of 8 weeks Inpatient and community	Randomised to adjunctive treatment with: A. Ondansetron 8 mg/day (*N* = 15) B. Placebo (*N* = 15) *N* = 30, 12 weeks	A. 33.00 ± 5.88 B. 33.53 ± 5.95 11/30 (37%) female	Primary outcome: PANSS Mean, total and subtotal scores Secondary outcomes: WMS-R: Visual Paired Associates 1&2, Visual Reproduction 1&2, Block design	Student’s *t* test Fisher’s exact test	Significant improvement in PANSS total score at 12 weeks (*t*_28_ = 6.65, *p* < 0.001) Ondansetron associated with improvement on Visual Reproduction 1 (*p* = 0.05) and 2 (*p* = 0.05), Visual Paired Associates 1 (*p* = 0.04) and 2 (*p* = 0.04)
Hall, 1991 UK Double-blind placebo-controlled crossover study	Healthy male volunteers NR	Randomised in terms of order: A. Ondansetron 1 mg BD B. Ondansetron 8 mg BD C. Placebo BD D. Placebo BD for 2 days + final dose of lorazepam 2 mg *N* = 12, 2.5 days	31 (range 21–40) 0/12 (0%) female	Primary outcomes: CRT Simulated car tracking	Trapezoidal integration. Analysis of covariance	No effect of ondansetron on CRT or simulated car tracking
Koike, 2005 Japan Cohort study	Diagnosis of schizophrenia (DSM-IV NR	Tropisetron 10 mg administered to: A. Patients with schizophrenia (*N* = 22) B. Healthy controls (*N* = 15) *N* = 37, Single dose	39.8 ± 14 years 12/37 (32%) female	Primary outcome: P50 auditory invoked potentials, assessed using the paired-click paradigm (500 ms apart), and indexed by the T/C ratio.	Paired *t* test	Tropisetron improved P50 auditory gating in patients with schizophrenia (*t*_18_ = 2.32, *p* = 0.033) *only in non-smokers Change in P50 T/C ratio correlated with the baseline value (*r* = 0.85, *t* = 6.60, df = 17, *p* < 0.001),
Leigh, 1991 UK Double-blind placebo-controlled crossover design	Healthy male volunteers NR	Randomised in terms of order: A. Placebo capsule + placebo infusion B. Placebo capsule + granisetron 160 mcg/kg infusion C. Lorazepam 2.5 mg capsule + placebo infusion D. Lorazepam 2.5 mg capsule + granisetron 160 mcg/kg infusion *N* = 12, 1 day	32 (range 19–46) 0/12 (0%) females	Primary outcomes: CRT RVP (non-standardised)	Repeated-measures ANOVA. Greenhouse-Geisser correction	Granisetron associated with a reduced mean latency of correct responses on RVP (main effect, *p* = 0.03) No change in CRT
Levkovitz, 2005 Israel Double-blind placebo-controlled crossover trial	Diagnosis of schizophrenia (DSM-IV), who had been in remission for 6 months, and treated with fixed doses of clozapine (360 mg/day) Community	Randomised in terms of order: A. Ondansetron (8 mg OD) B. Placebo *N* = 21, 7 days	31.2 ± 8.9 0/21 (0%) female	Co-primary outcomes: PANSS, Mean, total and subtotal scores WAIS-R: Digit Span, Digit Symbol, Paired association, Rey–Osterreich Complex Figure	Repeated-measures ANOVA Paired Student’s *t* tests	No change in PANSS total or subtotal scores. Ondansetron improved Rey–Osterreich performance (*p* = 0.002). No effect on other subtests
Samandi, 2017 Iran Double-blind placebo-controlled parallel group trial	Diagnosis of schizophrenia (DSM‑IV‑TR), treatment-resistant, stable on risperidone (4–6 mg/d) for at least 2 months prior to enrolment. Inpatient and community	Randomised to adjunctive treatment with: A. Ondansetron 4–8 mg/day (adjunct to risperidone 8 mg/day) (*N* = 18) B. Placebo (adjunct to risperidone 8 mg/day (*N* = 20) *N* = 38, 12 weeks	A. 40 (CI: 37.43 to 43.26) B. 37 (CI: 34.02 to 39.20) 3/38 (7.9%) female	Primary outcome: PANSS mean, total and subtotal scores Secondary outcomes: WAIS-R: Picture Completion Object assembly Comprehension	Parametric and nonparametric methods. Student’s *t*-test. Chi-squared	Ondansetron associated with reduction in PANSS positive and negative scores (*p* < 0.001). Ondansetron associated with improved performance on Object assembly (*p* < 0.001) and Comprehension (*p* < 0.001).
Shiina, 2010 Japan Randomised placebo-controlled parallel group trial	Diagnosis of schizophrenia, (DSM-IV-TR), stable on risperidone (2–6 mg/day) for minimum 8 weeks. Community setting	Randomised to adjunctive treatment with: A. Tropisetron B. 10 mg/day (*N* = 20) C. Placebo (*N* = 20) *N* = 40, 8 weeks	A. 34.96 ± 6.82 11/20(55%) female B. 35.15 ± 8.54 10/20(50%) female 21/40(53%) female	Co-primary outcomes: P50 auditory invoked potentials, assessed using the paired-click paradigm (500 ms apart), and indexed by the T/C ratio. CANTAB (RVP, DMS), PANSS	Chi-squared. ANOVA. Paired *t* test. Bonferroni corrections for multiple CANTAB comparisons. Sensitivity analysis (whole sample and non-smokers only)	Tropisetron *t*_15_ = 3.24, *p* = 0.006), but not placebo (*t*_13_ = 0.570, *p* = 0.6), improved P50 auditory gating deficits. Tropisetron improved RVP performance accuracy but only in non-smokers, after Bonferroni correction (*t*_11_ = 5.78, *p* < 0.001). No change in PANSS.
Stern, 2019 USA Double-blind placebo-controlled crossover trial	Healthy population Advertisements posted on the internet and university campus	Randomised in terms of dose and order: A. Ondansetron 8 mg + placebo (*N* = 19) B. Ondansetron 16 mg + Placebo (*N* = 21) C. Ondansetron 24 mg + Placebo (*N* = 18) *N* = 58, Single dose	A. 32 ± 11 B. 29 ± 8 C. 33 ± 11 25/53 (47%) female	Primary Outcomes: fMRI activation following body BFV and CV tasks	Regression Analysis	Ondansetron 24 mg associated with reduced activation in insula, sensorimotor cortex premotor areas, ACC, and temporal cortex relative to placebo during CV video task. No differences observed during BFV tasks.
Xia, 2020 China Double-blind placebo-controlled parallel group trial	Diagnosis of schizophrenia (DSM-IV) schizophrenia stable on risperidone (3–6 mg/dd) for at least 1 month prior to participation in the study Inpatient	Randomised to adjunctive treatment with: A. Tropisetron 5 mg/d B. Tropisetron 10 mg/d C. Tropisetron 20 mg/d D. Placebo *N* = 3, 1 day	A. 29.60 ± 8.90 B. 26.30 ± 5.25 C. 31.50 ± 9.93 D. 36.50 ± 10.61 9/38 (23.7%) female	Co-primary Outcomes: P50 evoked potential 1 h after taking tropisetron or placebo RBANS	MANOVA: Age, education, gender, illness duration, dose as covariates if interactive effect of drug × time was significant. ANCOVA/Dunnett test used to compare doses. Bonferroni correction.	Main effect of tropisetron on P50 ratio (F_3,29_ = 3.39, *p* < 0.05), but did not survive Bonferroni correction. Main effect of tropisetron on RBANS total (F_3,29_ = 3.81, *p* < 0.05), and immediate memory (F_3,29_ = 3.92, *p* < 0.05) but not visuospatial subtests. No differences observed between doses.
Zhang, 2012 China Double-blind placebo-controlled parallel group trial	Diagnosis of schizophrenia (DSM-IV), non-smoking patients stable on risperidone 3–6 mg/day at least 1 month prior to enrolment Inpatient	Randomised to adjunctive treatment with: A. Tropisetron 5 mg/day (*N* = 10) B. Tropisetron 10 mg/day (*N* = 10) C. Tropisetron 20 mg/day (*N* = 10) D. Placebo (*N* = 10) *N* = 40, 10 days	Range 20–55 10/40 (25%) female	Co-primary outcome: P50 auditory invoked potentials, assessed using the paired-click paradigm (500 ms apart), and indexed by the T/C ratio. RBANS	MANOVA, ANCOVA. Chi-squared. Bonferroni corrections. Controlled for confounding effects of age, education, sex, illness duration, risperidone dose, chloral hydrate use.	Tropisetron reduced P50 gating ratio (F_3,28_ = 7.31, *p* = 0.001). No differences observed between doses. Drug-by-time effect on RBANS total (F_3,28_ = 4.20, *p* = 0.01) and immediate memory (F_3,28_ = 3.43, *p* = 0.03) but not visuospatial subtests. Correlation between improved immediate memory and reduced gating ratio (*r* = 0.38, *p* < 0.01)

*NR* not reported; *RBANS* Repeatable Battery for the Assessment of Neuropsychological Status; *CANTAB* Cambridge Neuropsychological Test Automated Battery; *DMS* delayed matching to sample; *RVP* Rapid Visual information Processing; *CRT* choice reaction time; *WAIS-R* Wechsler Adult Intelligence Scale – Revised.

### Outcome measures

Outcome measures used in the included studies are described in Table 2. Eight studies were carried out in patients with schizophrenia who were symptomatically stable on antipsychotic medication. Five studies measured the impact of setrons on sensory gating, using the P50 electroencephalogram (EEG) event-related potential (ERP) waveform response to a repeated auditory stimulus [31, 32, 34, 35, 40]. The P50 waveform response occurs 50 ms after an auditory stimulus. In the paired-click paradigm, the initial stimulus (S1) is followed by a second stimulus (S2) 500 ms later, and evidence of ‘gating’ is shown by a reduction in amplitude of S2. In healthy participants the S2/S1 ratio is <0.5, but is larger in patients with schizophrenia [24–26]. Three of the studies included measures of visuoperceptual function (as part of a wider battery of neuropsychological tests) as co-primary outcomes [31, 32, 34]. Three studies included measures of visual processing as a primary outcome [38] or as secondary outcomes in studies whose primary aim was to investigate the effect on adjunctive ondansetron on the Positive and Negative Symptom Scale (PANSS) total and subtotal scores [29, 33]. Three studies, carried out in healthy participants, included measures of visuoperceptual [36, 37] and sensorimotor processing [39] as primary outcome measures.

**Table 2:** Outcome measures.

Author, year	Test	Brief description
Adler, 2005 Koike, 2005 Shiina, 2010 Xia, 2020 Zhang, 2012	*P50 Sensory Gating* Electroencephalogram (EEG)	Tests sensory gating using the P50 Event-related potential (ERP) waveform paired-click paradigm. The P50 waveform is an event-related potential measured on EEG, occurring 50 ms after an auditory stimulus. The participant is presented with two auditory clicks: S1 (Control, C) and S2 (Test, T), presented within 500 ms of each other. Evidence of ‘gating’ (attenuation of the wave) can be seen in the second P50 wave. Gating is calculated using of a composite score which relates S1 and S2 amplitudes either as ratios (T/C ratio, suppression ratio, S2/S1 ratio) or differences (S1 − S2).
Shiina, 2010	*Cambridge Neuropsychological Test Automated Battery* *(CANTAB)* Computerised non-verbal test battery	Subtests of interest: *Rapid Visual Information Processing*: A white box is shown in the centre of the screen, inside which digits from 2 to 9 appear in a pseudo-random order, at the rate of 100 digits per minute. Participants are requested to detect target sequences of digits. Outcome measures: accuracy, (number of correct responses), latency (speed of response), probability of false alarms and sensitivity. *Delayed Match to Sample*: An abstract and non-verbal sample is presented to the participants. After a short period of time, they are also presented four similar patterns. The participants have to choose the pattern that exactly matches the sample. Outcome measures: accuracy and latency. *Choice Reaction Time*: An arrow-shape appears on either side of a screen on which there are two buttons, one on either side. Participants press the appropriate button depending on where the arrow appears. Outcome measures: accuracy and latency, errors of commission and omission (late and early responses). *Pattern Recognition Memory*: A series of patterns are shown, and participant tasked with discriminating between new and repeated patterns. Outcome measures: accuracy and latency. *Spatial Recognition Memory*: A series of squares located on a screen are shown. Participant is tasked with remembering previous positions of the square. Outcome measures: accuracy and latency. *Spatial Span*: Participant presented with white square that changes colour. Participant tasked with remembering the order of colour change. Outcome measure: span length of order. *Spatial Working Memory*: Participant must use a process of elimination to uncover squares to find the target square. Outcome measure: accuracy. *Stockings of Cambridge*: Participant tasked with rearranging images which can be done in a discreet number of moves. Outcome measures: difficulty level reached, mean moves used and thinking time *Intra-extra Dimensional Set Shifting*: Participant tasked with determining a ‘rule’ that determines which of two visual stimuli consisting of shapes is ‘correct’ based on feedback. Outcome measures: errors, attempts, stages completed, latency
Leigh, 1991	*Rapid Visual Information Processing* (non-standardised version) Computerised non-verbal test of psychomotor funtion	Five different letters of the alphabet presented on the screen in a pseudo-random sequence, and in both lower and upper cases (A, B, D, E and H); 400 presentations/rate of one per second, on display for 0.1 s. Participants were required to press a response button when they identified consecutive presentations of the same letter, irrespective of case. Outcome measures: accuracy and latency.
Leigh, 1991	C*hoice Reaction Time* (non-standardised version) Computerised non-verbal test of psychomotor funtion	Two buttons with neighbouring LEDs (light-emitting diodes) are equally-spaced with a control button. When an LED lights, participants transfer a digit from the control button to the colourful button next to the lit LED and afterwards return the digit to the control button until one of the two LEDs lights again. During this test participants are presented with concurrent auditory misdirection. Outcome measures: accuracy (number of correct responses) and latency (reaction time and movement time)
Hall, 1991	*Choice Reaction Time* (Leeds Psychomotor tester) Computerised non-verbal test of psychomotor funtion	Six red lights shine randomly one at the time. Participants have to turn off the light light by pressing a corresponding button. Outcome measure: time taken to respond to the red light stimulus together and total time taken to both respond and cancel the light.
Hall, 1991	*Simulated Car Tracking* Computerised non-verbal test of psychomotor function	A computer-based test where participants had to maintain the cursor in line with a moving target using a joystick. Outcome measures: accuracy of tracking, response time to 10 peripherally presented visual stimuli recorded.
Xia, 2020 Zhang, 2012	*Repeatable Battery for neuropsychological status (RBANS)* Non-computerised cognitive screening battery	Subtest of interest: *Figure Copy*: Participant asked to copy a geometric design consisting of 10 parts. Outcome measure: accuracy *Line Orientation*: Participant asked to match a presented line in terms of orientation, to a group of 13 lines spanning 180 degrees of orientation. Outcome measure: accuracy Other subtests as part of RBANS performed by both Xia and Zhang: *List Learning*: Recall accuracy of 10-item list *Story Memory*: Recall accuracy of 12-item story *Picture Naming*: Accuracy of picture naming line drawings *Semantic Fluency*: Total number exemplars named in 60 s *Digit Span*: Ability to recall span of digits presented sequentially *Digit Symbol*: Accuracy of matching symbols from a table of corresponding digit/symbol pairs *Delayed Memory*: Recall of elements of previous subtests
Akhondzadeh, 2009	*WMS-R: Wechsler Memory Scale – Revised* *(WMS-R)* Non-computerised memory test battery	Subtests of interest: *Visual Paired Associates 1*: Participants required to remember the colour that was linked with each of six abstract line drawings. *Visual Paired Associates 2*: Delayed-recall trial administered (half an hour) after Visual Paired Associates 1 *Visual Reproduction 1*: Participant is shown a design for 10 s. After the removal of the design, they had to draw it from memory (3 trials) *Visual Reproduction 2*: Delayed-recall trial administered (half an hour) after Visual Reproduction 1 *Picture Completion*: Participant is required to select the missing part of a picture. *Figural Memory*: Participant required to identify the correct figure Other Subtests performed by Akhonzadeh: *Verbal Paired Associates*: Recall accuracy of words paired with other words *Logical Memory*: Ability to recall narrative features of a section of prose *Digit Span*: Ability to recall span of digits presented sequentially
Levkovitz, 2005 Samadi, 2017 Akhonzadeh, 2009	*Wechsler Adult Intelligence Scale –Revised* *(WAIS-R)* Non-computerised IQ test battery	Subtests of interest performed by Levkovitz: *Digit Span*: Ability to recall span of digits presented sequentially *Digit Symbol*: Participant required to match a list of digits to its corresponding symbol from a table of digit/symbol pairs. Subtests of interest performed by Samadi: *Picture Completion*: Participant is shown recognisable image with large section missing, and is asked to state what is missing *Object Assembly*: Participant is required to put together puzzle pieces to make different objects. Other subtests performed by Samadi *Information*: Participant is asked general knowledge questions *Verbal Comprehension*: Participant is asked to identify the qualitative relationship between words Subtest of interest performed by Akhonzadeh: *Block Design*: Participant is required to restructure blocks with different colour patterns on different sides to match a pattern Other WAIS-R subtests: *Vocabulary*: Participant asked to define 30 words *Arithmetic*: Participant asked to solve arithmetic problems
Levkovitz 2005	*Rey–Osterrieth Complex figure Test* Non-computerised test of visuospatial and working memory	Participant is presented an 18-item complex drawn figure and asked to draw it from memory. Outcome measure: accuracy
Stern, 2019	*Body Focused video Task* fMRI	Participant is shown two types of video: ‘Body Focused’ videos (e.g. the tip of a brush stroking a hand) designed to elicit activation of brain areas associated with interoception and corresponding ‘Control’ videos (e.g. the tip of a brush moving across a table). Participants tasked with counting the number of repetitions of the given action shown in the video e.g. brush strokes. Outcome measure: Differences in fMRI activity between the two video types are assessed.
Akhonzadeh, 2009 Levkovitz, 2005 Samandi, 2017 Shiina, 2010	*Positive and Negative Symptom Scale* *(PANSS)* Clinician administered symptom scoring scale	Subjective scale administered by clinician following semi-structured interview. Severity measures across 7 items in Positive symptoms scale and 7 items in Negative symptom scale and 16 items across General Psychopathology scale.

This table describes the tests and subtests used amongst the studies. Some papers used discreet subtests from a Battery. For clarity, all subtests from a battery are listed here, and the subtests used by a given paper are then described. The subtest of interest to our review are stated explicitly.

### Quality assessment

Three studies were assessed as being of high quality [29, 32, 34], five studies were assessed as being of moderate quality [31, 33, 35, 36, 39] and three studies were assessed as being of low quality [37, 38, 40]. Quality assessment ratings are shown in Table 3.

**Table 3:** Quality assessment.

Author & date	Title	Selection bias	Study design	Confounders	Blinding	Data collection methods	Withdrawals and drop-outs	Global rating 1 = strong 2 = moderate 3 = weak
Adler 2005	Improved P50 Auditory Gating With Ondansetron in Medicated Schizophrenia Patients	3	1	1	1	1	1	2
Akhondzadeh 2009	Added ondansetron for stable schizophrenia: a double-blind, placebo-controlled trial	1	1	1	1	1	1	1
Hall 1991	A study to evaluate the effect of ondansetron on psychomotor performance after repeated oral dosing in healthy subjects	3	1	1	1	1	2	2
Leigh 1991	Effects of granisetron and lorazepam, alone and in combination, on psychometric performance	3	1	1	2	1	3	3
Levkovitz 2005	The effect of Ondansetron on memory in schizophrenic patients	3	1	3	3	1	3	3
Koike 2005	Tropisetron improves deficits in auditory P50 suppression in schizophrenia	2	2	3	3	1	3	3
Samadi 2017	Efficacy of Risperidone Augmentation with Ondansetron in the Treatment of Negative and Depressive Symptoms in Schizophrenia: A Randomized Clinical Trial	2	1	3	1	1	1	2
Shiina 2010	A randomised, double-blind, placebo-controlled trial of tropisetron in patients with schizophrenia	1	1	1	1	1	1	1
Stern 2009	High-dose ondansetron reduces activation of interoceptive and sensorimotor brain regions	3	1	1	2	2	1	2
Xia 2020	One-day tropisetron treatment improves cognitive deficits and P50 inhibition deficits in schizophrenia	2	1	1	1	1	1	1
Zhang 2012	Short-Term Tropisetron Treatment and Cognitive and P50 Auditory Gating Deficits in Schizophrenia	3	1	1	1	1	1	2

### Findings

Findings from the included studies are described below and are also detailed in Table 1.

### Sensory gating

The effect of a single 16 mg dose of ondansetron on P50 gating was investigated using a placebo-controlled crossover design in eight patients [35], seven of whom had been prescribed unspecified first generation (dopamine D2/3 selective) antipsychotics and one olanzapine (second-generation antipsychotic with 5HT3 antagonist properties). Evoked potentials were measured 1, 2 and 3 h post treatment, to coincide with peak plasma concentrations. There was a highly significant reduction in the S2/S1 ratio following ondansetron, which achieved a maximum at two hours post treatment, the time of peak plasma concentration (ondansetron ratio 41.4%; placebo mean = 80.2%).

Tropisetron was evaluated in four studies [31, 32, 34, 40]. The first [40] was described as a proof of principle study, which aimed to investigate the effects of a single 10 mg dose of tropisetron on sensory gating deficits in 22 patients with schizophrenia who were symptomatically stable on a range of antipsychotics, and in 15 healthy volunteers. A significant improvement in sensory gating was observed, but only in patients who were non-smokers. The authors suggested that future studies should recruit only non-smokers, to avoid the potential confounding effects of long-term nicotine exposure on α7-nicotinic receptors.

Three subsequent studies used placebo-controlled designs to evaluate the impact of tropisetron on patients who were stable on risperidone (2–6 mg daily), which was chosen because it does not target α7-nicotinic or 5HT3 receptors [31, 32, 34]. The studies differed in relation to their number of treatment arms, administered doses, treatment duration, and the inclusion or exclusion of smokers. One study [32], which was rated as high quality, compared tropisetron 10 mg/day to placebo over 8 weeks in 40 patients (20 per arm; 5 smokers in the placebo and 6 in the tropisetron arm) and found a reversal of P50 gating deficits in the tropisetron, but not placebo group, that was restricted to non-smokers. Tropisetron was also superior to placebo in improving the accuracy of performance on the Rapid Visual Information Processing (RVP) task of visual sustained attention [41], but only in non-smokers. There was no significant correlation between the degree of change in S2/S1 ratio and RVP performance which the authors attributed to the small sample size.

Two studies restricted the sample to non-smokers and compared three doses of tropisetron (5 mg, 10 mg, 20 mg) to placebo (10 in each arm), either after single-dose administration [34] or 10 days treatment [31]. There was an overall effect of tropisetron on S2/S1 ratio, which did not survive correction for multiple comparisons in the single-dose study, and showed no difference between doses. Both studies investigated cognitive function using the Repeatable Battery for Neuropsychological Status RBANS; a brief neuropsychological screening battery that includes 12 cognitive domains (language, attention, immediate memory, visuospatial/constructional and delayed memory) [42], and found a significant drug-by-time effect on RBANS total and the immediate (verbal) memory subtotal. The study that evaluated the effect of 10 days treatment observed a correlation between the extent of the reduction in P50 gating ratio and improved performance on immediate memory [31].

### Visuoperceptual function

Two placebo-controlled trials, whose primary aim was to evaluate the effect of ondansetron as an adjunctive treatment for cognitive and negative symptoms in patients with chronically stable schizophrenia who were treated with risperidone, included tests of visuoperceptual function as secondary outcomes. These studies reported a reduction in PANSS total [29] and on positive and negative subscores [33], respectively, in ondansetron treated patients. There were also improvements in performance on tests of visual memory (visual reproduction, visual paired associate learning) [29] and visuoperceptual (object assembly) ability [33]. Neither study corrected for statistical effects of the multiple tests included in the respective batteries.

One placebo-controlled trial evaluated 7 days adjunctive treatment with ondansetron on co-primary outcomes (PANSS and visuoperceptual and spatial function) in patients treated with clozapine who had been in symptomatic remission for 6 months [38]. No effect was observed in PANSS scores. Visual memory performance (Rey Osterrieth complex figure test) improved in the ondansetron treated group, but no differences were found in relation to other tests (digit span, digit symbol, paired associates).

A single placebo-controlled crossover study evaluated the effects of granisetron, alone and with lorazepam, in 12 healthy participants [37]. Granisetron reduced the latency of performance on a test of visual sustained attention but did not improve performance accuracy and showed no effect on choice reaction time. No effects of ondansetron were observed on choice reaction time or simulated car tracking tests [36].

### Interoception

One study used functional MRI to evaluate effects of ondansetron on interoception (the ability to interpret internal sensations such as satiety, respiration, heartbeat and relate them to emotions) [39]. The study was hypothesis-driven, based on ondansetron’s use to decrease pruritis and pain. Fifty-three participants were randomized (by order and dose) to receive a single dose of ondansetron or placebo before carrying out an fMRI task previously shown to engage interoceptive circuitry. The highest dose (24 mg) of ondansetron reduced activation in the interoceptive circuit (insula, sensorimotor cortex, premotor area, anterior cingulate cortex and temporal cortex), but only when participants were viewing the control video.

## Discussion

Previous meta-analyses of 5HT3 antagonists have reported on the efficacy of ondansetron as an adjunctive treatment for negative symptoms and general psychopathology in patients with chronic, stable schizophrenia [21]. In this review, we focused specifically on the impact of ondansetron and other 5HT3 antagonists on sensory processing, to establish whether preclinical findings of improved sensory gating would translate to improvements in humans that could explain the mechanism of antipsychotic treatment effects.

The most consistent finding was that ondansetron and tropisetron were superior to placebo in reversing impairments in P50 suppression in patients with schizophrenia, when administered as an adjunct to existing medication. This effect was observed following a single dose of ondansetron, and after single-dose and steady-state treatment with tropisetron. Tropisetron also improved sustained visual attention in a single study. There was inconsistent evidence of an effect of ondansetron other tests of visuoperceptual processing in patients with schizophrenia, and no evidence that ondansetron or granisetron improved performance on test of visuoperceptual performance in healthy participants.

Interpretation of the findings is limited by the small number of included studies, relatively small sample sizes, and methodological heterogeneity; including in the population studied (schizophrenia versus healthy participants); the lack of specificity of test performance measures, many of which were administered as part of larger, standardised batteries; and the confounding effects of antipsychotic or other psychotropic medication in patients with schizophrenia. It is also that possible that potential improvements on visuoperceptual processing might only be detected in patient groups with impaired visuoperceptual processing. This might explain the absence of any effects in healthy participants in the studies reviewed. Perhaps the most important limitation is the lack of placebo-controlled data on patients with untreated positive symptoms, which limited interpretation of the extent of ondansetron’s ‘antipsychotic’ effects.

Despite these limitations, there was consistent and robust evidence that 5HT3 antagonists enhance sensory processing in humans, especially when assessed using neurophysiological markers. Impaired sensory gating reflects a reduced ability to filter irrelevant information [43] and is considered a robust neurophysiological marker of psychosis in schizophrenia and bipolar disorder [44]. It has been suggested that an abnormal P50 ratio represents a neurocognitive deficit and vulnerability marker that is present across the psychosis spectrum [24–26], characterised by poorer performance on tests of sustained attention and vigilance [26, 45], including greater noise-induced distractibility during attentional tasks. The S2/S1 ratio of the P50 waveform response has been associated with the ‘global inattention’ scale of the Scale for Assessment of Negative Symptoms (SANS) [46] and with trait severity of auditory hallucinations, measured by the Psychotic Symptoms Ratings Scale (PSYRATS) [47].

Auditory gating is modulated through interplay between serotonergic, cholinergic and GABAerbic neurotransmission [44]. Cholinergic inputs from the septal nucleus interact with 5HT3-expressing GABAergic interneurons in the CA3-CA4 hippocampal regions during the early phases of auditory processing, leading to transient inhibition of pyramidal neurones and suppression or gating of the response to a repeated stimulus [48]. Hippocampal α7-nicotinic receptors have been implicated in this process [49], based on the finding that α7-nicotinic receptor antagonists block both ondansetron and tropisetron’s reversal of gating deficits in DBA/2 mice [14, 16, 50]. Genetic studies carried out in people with schizophrenia and their families have similarly linked gating deficits to the α7 subunit of the nicotinic cholinergic receptor gene [51, 52].

Sensory gating is a ‘bottom up’ filter but is also influenced by top down processes [53, 54]. Studies that have used invasive techniques in animal and human neuroimaging studies have implicated a widely distributed network that spans temporoparietal and prefrontal regions [48] and emphasised the importance of synchronised neuronal firing in maintaining efficient cognitive and perceptual processing [55]. Cortical GABAergic inter-neurones are important in this respect, as they facilitate sensory integration and modulate functional network dynamics [11]. The thalamus acts as a gateway for sensory (feedforward) inputs and a hub for cortical feedback, which is then relayed to GABAergic inter-neurones in the supragranular layer L1. In vitro studies have shown that 30% of GABAergic inter-neurones in the primary somatosensory cortex express 5HT3-A receptors, the majority of which are located in L1; an optimal location for their role in modulating cortical sensory processes [56]. Cholinergic activation acts to further increase the precision of sensory input processing [57] by increasing the signal-to-noise ratio of cortical neuronal responses to sensory inputs [58]; with nicotinic receptors, expressed in the hippocampus, thalamic reticular nucleus, and geniculate nuclei [59] playing a key role in this process [60].

The relative importance of the contribution of α7-nicotinic partial agonist, versus 5HT3 antagonist mediated effects, on sensory gating is unclear. Research in DBA/2 mice has shown differential effects of the α7-nicotinic receptor partial agonist DXMB-A (reduced S2 amplitude), the full α7 agonist varenicline (which has no effect on sensory gating) [61], and the selective 5HT3 receptor antagonists ondansetron and tropisetron (reduced S2 and increased S1 amplitude) [12, 14]. These findings suggest that 5HT3 antagonist effects alone are sufficient to reverse gating deficits, and that full α7 receptor agonism is not beneficial, possibly due to receptor desensitisation [32].

A direct comparison of the neurophysiological effects of ondansetron and tropisetron, and their relationship with changes in neuropsychological test performance and clinical symptoms would provide a mechanistic understanding of treatment effects. Future studies should also consider including drugs such as encenicline, an α7-nicotinic receptor partial agonist with 5HT3 antagonist effects [62, 63], and CVN058, a novel selective 5HT3 antagonist [64], which are also of interest as adjunctive treatments for patients with schizophrenia.

The effects of 5HT3 antagonists on the RVP test should be further explored, as impaired test performance is viewed as an endophenotypic marker of cognitive vulnerability to psychosis [65–67] and, importantly, the test has proved sensitive to the effects of tropisetron. This area of research could also be extended to include antipsychotic naive patients with prodromal or milder psychotic symptoms, to provide additional insights into the mechanisms of any treatment effects at an early stage of the illness.

From a neurochemical perspective, the modulatory effects of 5HT3 antagonists on nicotinic cholinergic and GABAergic neurotransmission are highly relevant when considering the treatment of hallucinations in PD, as there is marked disruption of nicotinic cholinergic neurotransmission in people with PD [68], and magnetic resonance spectroscopy (MRS) has shown a reduction in GABAergic activity in the occipital cortex in PD patients who hallucinate compared to those without [69]. The improvement in visual sustained attention following the use of 5HT3 receptor antagonists also warrants further consideration, as integrative theories regarding the origins of misperceptions and hallucinations propose that they arise as a result of impairments in visual perception and attentional binding [70]. Aligned with this, the Activation, Input, Modulation model proposes that hallucinations occur as a result of dysregulation of gating and filtering of sensory inputs, which reduces the dominance of inputs from external sources and increases cortical responses to internally generated imagery [71]. The fact that people with PD who experience hallucinations tend to over-rely on prior knowledge compared to those who do not [72] supports these theories. Auditory gating deficits have been observed in patients with PD compared to healthy subjects; especially in patients with more severe disease (Hoehn and Stages IV–V). However, there has been no direct comparison of hallucinators versus non-hallucinators.

The TOP HAT Trial (Clinical trials.gov NCT04167813) will provide placebo-controlled data on the effectiveness of ondansetron as a treatment for hallucinations in PD. Further research is, however, needed to investigate the relationship between sensory gating, visual information processing and emergent hallucinations in PD. Interventional studies are also needed to compare the effects of ondansetron and tropisetron (or drugs with similar properties) on neurophysiological, visuoperceptual and imaging markers of hallucinations.

## Supplementary information

PRISMA flow diagram
