# Supplementary material for: Selective 5HT3 antagonists and sensory processing: a systematic review
Source: Neuropsychopharmacology. 2022 Jan 11;47(4):880–90. doi: 10.1038/s41386-021-01255-4 (PMC8882165; doi:10.1038/s41386-021-01255-4)
Supplement: Supplementary file 1 — PRISMA flow diagram [file 41386_2021_1255_MOESM1_ESM.doc]

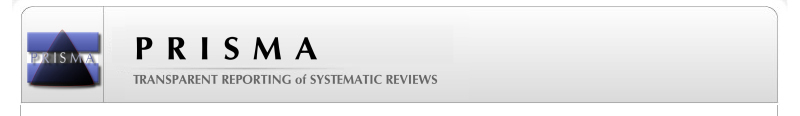
**PRISMA 2009 Flow Diagram**

**Screening**

**Included**

**Eligibility**

**Identification**

Records identified through database searching
(n = 2,162)

Additional records identified through other sources
(n =3)

Records after duplicates removed
(n = 2, 040)

Records screened
(n = 2,040)

Records excluded
(n = 2, 026)

Full-text articles assessed for eligibility
(n =14 )

Full-text articles excluded, with reasons
(n = 3)

1: Conference Abstract

2: Unable to locate full text

3: Functional duplicate

3:

Studies included in quantitative synthesis
(n = 11)
